# CD4^+^ T Cell Profile and Activation Response in Sickle Cell Disease Patients with Osteonecrosis

**DOI:** 10.1155/2020/1747894

**Published:** 2020-10-09

**Authors:** Paula B. Daltro, Tiago O. Ribeiro, Gildásio C. Daltro, Roberto J. Meyer, Vitor Fortuna

**Affiliations:** ^1^Health Science Institute, Federal University of Bahia, Salvador, BA 40110-100, Brazil; ^2^Prof. Edgar Santos Hospital Complex, HUPES, Federal University of Bahia, Salvador, BA 40110-060, Brazil

## Abstract

Recent evidence suggests that abnormalities involving CD4^+^T lymphocytes are associated with the pathophysiology of osteonecrosis (ON); however, few studies have addressed the CD4^+^T cells in ON related to sickle cell disease (SCD/ON). In addition, T cells producing multiple cytokines simultaneously are often present in the inflammatory milieu and may be implicated in the immune response observed in SCD/ON. In the present study, we aimed to characterize the functional status of CD4^+^T cells in SCD by simultaneously determining the frequency of IFN-*γ*^+^, IL-4^+^, and IL-17^+^ CD4^+^T in cell cultures under exogenous stimuli. Peripheral blood mononuclear cells (PB-MNCs) from 9 steady-state SCD patients, 15 SCD/ON patients, and 19 healthy controls had functional status of CD4^+^T cells analyzed. Bone marrow mononuclear cells (BM-MNCs) from 24 SCD/ON patients (SCD BM) and 18 patients with ON not related to SCD (non-SCD BM) were also analyzed. We found that PB-MNC of SCD patients with or without ON presented significantly reduced TCD4^+^, TCD8^+^, and TCD4^+^ naïve cell frequencies and increased frequency of circulating CD4^+^T cells able to simultaneously produce IFN-*γ*^+^/IL4^+^ and IL-17^+^/IL4^+^ compared to healthy controls. Conversely, the polyclonal stimulation of BM-MNC induced an increased frequency of CD4^+^IFN-*γ*^+^ and CD4^+^IL-17^+^ in SCD BM compared to non-SCD BM. The increased proportion of CD4^+^ T cells able to produce a broad spectrum of proinflammatory cytokines after a strong stimulus indicates that the immune system in SCD/ON patients presents an expressive pool of partially differentiated cells ready to take on effector function. It is possible that this increased subpopulation may extend to inflammatory sites of target organs and may contribute to the maintenance of inflammation and the pathophysiology of osteonecrosis in sickle cell disease.

## 1. Introduction

Sickle cell disease (SCD) is caused by a point mutation in the *β*-globin gene (HBB), resulting in the production of hemoglobin S (HbS). The abnormal hemoglobin polymerizes upon deoxygenation and produces deformed red blood cells (RBCs). This primary pathophysiological alteration has many downstream effects including the production of several inflammatory molecules and responses that ultimately lead to hemolysis and vasoocclusion of microvessels [1]. Repeated microvascular occlusion, tissue hypoxia, and cytokine production perpetuate the chronic inflammatory state with a pivotal role in the vaso-occlusive crisis (VOC) and painful episodes, the major cause of hospitalization in SCD patients [2]. Impaired leucocyte function, defective humoral, and cell-mediated immunities underlie the immunocompromised state of patients with SCD [3–5]. In addition to driving the vaso-occlusive processes, inflammatory responses are associated with numerous complications of the disease including osteonecrosis [6, 7].

Osteonecrosis is a long-term debilitating complication of SCD, representing an important cause of morbidity in SCD patients [8]. Osteonecrosis commonly affects the hip but may concurrently involve other joints, including the knee, shoulder, ankle, and spine [9, 10]. SCD patients usually start with silent bone lesions. If left untreated, the disorder gradually progresses and the majority of SCD patients will experience structural bone deterioration, collapse of the joint, and degenerative arthritis [11].

Recent studies have demonstrated that inflammatory processes are associated with the development and progression of osteonecrosis. Abnormal immune response, imbalanced immune cell subpopulations, and higher plasma levels of inflammatory cytokines in patients with osteonecrosis suggest that inflammation plays a crucial role in this disorder [12–14]. Moreover, chemokines and cytokines released by necrotic cells recruit inflammatory cells, inducing local and systemic immune response in patients with osteonecrosis [15–17]. However, whether these inflammatory processes are also involved in SCD osteonecrosis has rarely been investigated.

To date, a few preliminary works have suggested alterations of the adaptive immune system and related cytokines in the development or progression of osteonecrosis in SCD patients [18]. Mukisi-Mukaza and coworkers described nonspecific granulomatous inflammatory reaction with a significant presence of lymphocytes in SCD osteonecrotic lesions [19]. Recently, Alagbe et al. demonstrated that SCD patients during bone infarction crisis have elevated TNF-*α*, IL-8, and ET-1 levels, which are critical mediators of inflammation, bone remodeling, and pain [20]. In addition, increased IL-6 levels, a pleiotropic cytokine produced during inflammatory response, were present in SCD patients with osteonecrosis in comparison to healthy SCD patients [6].

This study is aimed at investigating the possible association of T cell population in the pathophysiology of osteonecrosis that affects patients with sickle cell disease. We analyze peripheral blood and bone marrow aspirate for hematological aspects and peripheral blood mononuclear cells for CD4^+^, CD8^+^, CD45RA and CD45RO markers. We also investigated TCD4^+^-producing intracellular cytokines IFN-*γ*, Il-4, and Il-17 in PB-MNC and BM-MNC to better understand T cell response in osteonecrosis from SCD pathogenesis.

## 2. Materials and Methods

### 2.1. Participants

The institutional review board of the Health Science Institute (Federal University of Bahia-Salvador, Bahia-Brazil, protocol no. 67238317.0.0000.5662) approved this study. Written informed consent was obtained from all participants.

Between January 2017 and August 2019, peripheral blood (PB) was obtained from forty-three participants, both sexes, aged 10-55 years, and allocated in three groups: steady-state SCD patients (SCD, *N* = 9), SCD patients affected by ON (SCD/ON, *N* = 15), and healthy subjects (control *N* = 19). After enrollment, sociodemographic data and clinical information were collected. Exclusion criteria for these PB participants were recent viral infection, diabetes, autoimmune disease, immunodeficiency, pregnancy, obesity, presence of neoplastic disease, steroid treatment, acute pain/bone crisis, and hydroxyurea use. Sickle cell disease patients that experienced recurrent painful crises, displayed ulcers, or received blood transfusion at least three months before were also excluded.

Bone marrow (BM) aspirates were obtained from forty-two consecutive participants who underwent osteonecrosis treatment with autologous BM graft surgery, between August 2017 and September 2019. Enrolled participants, both sexes, aged 9-57 years, were allocated in two groups: participants affected by SCD and osteonecrosis (SCD, *N* = 24) and participants affected by osteonecrosis not related to SCD (non-SCD, *N* = 18). Inclusion criteria were precollapse ON, radiological diagnosis according to Ficat stage 0-IIA, and scoring at least 20 points on the pain and daily life activities questionnaire. The exclusion criteria were participants with Ficat stage III or higher, current or previous bone infection at the limb affected by necrosis, acute recurrent painful crises, recent viral infection, immunosuppressive drug therapy, a history of previous surgery on the same injured limb, pregnancy, and presence of neoplastic disease.

### 2.2. Peripheral Blood and Bone Marrow Collection

Peripheral blood (PB) samples were collected from the antecubital vein using heparin anticoagulant tubes (VACUETTE® Blood Collection Tubes–16/100). The bone marrow (BM) was harvested under general anesthesia from the posterior iliac crest of patients diagnosed with osteonecrosis. The bone marrow was aspirated in 20 mL heparinized plastic syringe. An automated analyzer (ABX Horiba/Pentra80) was used for the quantitative evaluation of hematological parameters.

### 2.3. Mononuclear Cell (MNC) Preparation

Peripheral blood (PB) and bone marrow aspirate (BMA) mononuclear cell (PB-MNC and BM-MNC, respectively) isolation was done using density gradient Ficoll-Paque™ (GE Healthcare) as described by the manufacturer.

### 2.4. Lymphocyte Subset Immunophenotyping Analysis

Immunophenotyping analysis was performed on PB-MNC and BM-MNC for the expression of cell surface antigens using direct four-color analysis with fluorescein isothiocyanate- (FITC-) conjugated, phycoerythrin- (PE-) conjugated, APC-conjugated, and peridinin chlorophyll protein complex- (PerCP-) conjugated monoclonal antibodies. Lymphocyte subsets were determined using BD Tritest CD3/CD4/CD8 and Multitest CD3/CD4/CD45RA/CD45RO (BD Biosciences, Brazil). In brief, approximately 250 thousand cells were labeled with 10 *μ*L of the fluorescent cocktail. After 30 min of incubation in the dark at room temperature, nonspecific binding was removed and cells were washed and analyzed immediately on a flow cytometer (BD FACS Calibur Flow Cytometer, BD Biosciences, Brazil), by selecting the appropriate area on the dot plot from the forward (FSC) and side scatter (SSC) histogram. The percentage of lymphocytes labeled with the investigated antigen was considered in the positive selection area.

### 2.5. Hematopoietic Progenitor Stem Cells (CD34^+^CD45^low^) Analysis

The frequency of hematopoietic stem cells (HSCs) and progenitor cells can be considered by CD34^+^CD45^low^ cell analysis [21]. For this purpose, BMA was submitted to CD34^+^CD45^low^ staining and analysis. Antibody reagent cocktail containing, anti-CD34 antibody (PE, Clone 581, mouse IgG1 *κ*, Exbio), anti-CD45 antibody (FITC, Clone 2D1, mouse IgG1 *κ*, Exbio), and 7-aminoactinomycin-D (7-AAD, BD Pharmingen) was added in 5 mL round-bottom tubes, immediately prior to use, one hundred microliters of BMA sample was added to each tube and incubated for 20 minutes in the dark at room temperature. Red blood cells (RBCs) were lysed (Excelllyse Easy; Exbio) followed by 10 minutes of incubation in the dark at room temperature. Flow cytometry data was acquired on a flow cytometer (BD FACS Calibur Flow Cytometer, BD Biosciences, Brazil), and analysis was performed using the Cell Quest software (BD Biosciences).

### 2.6. Intracellular Cytokine Profile Characterization of CD4^+^ T Cells

Intracellular cytokine profile was performed on PB-MNCs and BM-MNCs using the Human Th1/Th2/Th17 intracellular phenotyping kit (BD Biosciences, Brazil), according to the manufacturer's instructions. Approximately 1 million mononuclear cells/mL was incubated in RPMI 1640 (Sigma Aldrich, Brazil) medium, supplemented with 10% fetal calf serum, and stimulated for 5 hours with 50 ng/mL phorbol ester (PMA) and 1 *μ*g/mL calcium ionophore (Ionomicine), in the presence of the BD GolgiStop ™ Protein Transport Inhibitor. Nonstimulated cells were included as a control during the assay. After incubation, cells were washed, fixated, permeabilized, and stained with antibody cocktail containing, Human CD4 PerCP-Cy5.5 (clone: SK3); Human IL-17A PE (clone: N49-653); Human IFN-GMA FITC (clone: B27), and Human IL-4 APC (clone: MP4-25D2).

### 2.7. Statistical Analysis

Data were evaluated using descriptive statistics (mean, standard deviation, minimum, maximum, median, frequency, and ratio). The results were expressed as the mean (SD) for quantitative variables with normal distributions. The parameters of total and differential blood cell count according to distribution plots and analysis of variance homogeneity were presented as the median and the range (min-max). Student's *t*-test was used for intergroup comparison of normally distributed data. Comparison among more than two groups were carried out based on one-way ANOVA and post hoc Dunnett's test or Kruskal–Wallis tests. *p* values of < 0.05 were considered statistically significant. Statistical analysis was done using SPSS statistics (version 26) and GraphPad Prism (version 8) software.

## 3. Results

### 3.1. Patients with SCD Affected by Osteonecrosis Have Lower Hemoglobin and Higher WBC Count at Baseline Compared to Healthy Control Patients

The baseline demographic and PB hematological values are shown on Table 1. Demographic data was comparable among the groups, and no significant difference was observed. In comparison to the control group, patients with SCD and SCD/ON had a significantly lower median total red blood cell count (RBC) (5.4 (range 3.3-7.8) vs. 2.9 (range 2.5-5.1) and 3.7 (range 2.3 − 5.5) × 10^6^/mm^3^, *p* = 0.005), lower hematocrit (2.9 (range 2.5-5.1), 3.7 (range 2.3-5.5) vs. 5.4 (3.3 − 7.8) × 10^6^/mm^3^, *p* = 0.01 and *p* = 0.005), lower mean hemoglobin content (2.9 (range 2.5-5.1), 3.7 (range 2.3-5.5) vs. 5.4 (3.3 − 7.8) × 10^6^/mm^3^, *p* = 0.001 and 0.05), and higher mean corpuscular hemoglobin (MCH) (2.9 (range 2.5-5.1), 3.7 (range 2.3-5.5) vs. 5.4 (3.3 − 7.8) × 10^6^/mm^6^, *p* = 0.05) (Table 1). We also observed a significantly higher mean white blood cell count (WBC) in SCD/ON patients compared to the control group (9.6 (range 5.0-13.1) vs. 6.9 (3.5 − 9.3) × 10^3^/mm^3^, *p* = 0.0008). A similar trend to increased WBC count was noted in SCD patients but failed to reach statistical significance (7.4 (range 4.5-13.3) vs. 6.9 (3.5 − 9.3) × 10^3^/mm^3^, *p* = 0.182). While there was a relatively higher mean corpuscular volume (MCV) for patients with SCD compared to the control group (9.6 (range 5.0-13.1) vs. 6.9 (3.5 − 9.3) × 10^3^/mm^3^, *p* = 0.0008), there were no significant differences (NS) in total lymphocyte and platelet counts among the groups. The lack of significant difference in many baseline characteristics indicates similarities between the cohort of patients with SCD and SCD/ON evaluated here. In addition, these results demonstrate a need for better PB characterization of patients with SCD affected by osteonecrosis.

Bone marrow aspirated from 24 SCD patients affected by osteonecrosis (SCD BM) and 18 patients with osteonecrosis not related to SCD (non-SCD BM) were also analyzed. There were no significant differences in the distribution of age, disease duration, stages, and the values of index of femoral head collapse among the different groups of patients. While WBC cell count from SCD BM (*n* = 24) and non-SCD BM (*n* = 18) was similar (12.8 (range 6.2-31.9) vs. 7.8 (range 5.0 − 24.5) × 10^3^/mm^3^), RBC (12.8 (range 6.2-31.9) vs. 7.8 (range 5.0 − 24.5) × 10^3^/mm^3^, *p* = 0.01), and hemoglobin content (12.8 (range 6.2-31.9) vs. 7.8 (range 5.0 − 24.5) × 10^3^/mm^3^, *p* = 0.05) from SCD BM were significantly lower (Table 2). In contrast, SCD BM had significantly higher mean MCV (12.8 (range 6.2-31.9) vs. 7.8 (range 5.0 − 24.5) × 10^3^/mm^3^, *p* = 0.005), higher mean RDW (12.8 (range 6.2-31.9) vs. 7.8 (range 5.0 − 24.5) × 10^3^/mm^3^, *p* = 0.005), and higher median platelet counts (12.8 (range 6.2-31.9) vs. 7.8 (range 5.0 − 24.5) × 10^3^/mm^3^, *p* = 0.05) compared to non-SCD BM. The frequency of CD34^+^CD45^low^ was not significantly different between SCD BM and non-SCD BM (Table 2).

### 3.2. SCD Patients Have a Lower Proportion of TCD4^+^ and TCD8^+^ Cells Compared to Healthy Control Patients

**Figure 1:**
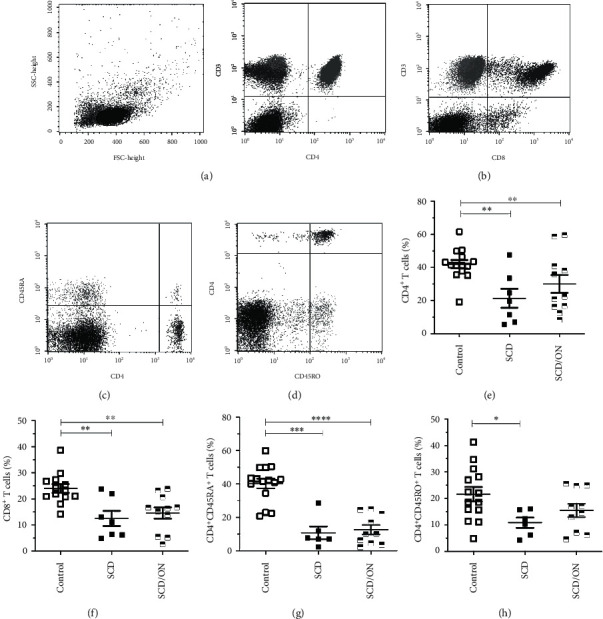
Relative frequency of major T cell populations in peripheral blood mononuclear cells from healthy donors (control), steady-state SCD, and SCD with osteonecrosis (SCD/ON) patients. Representative dot plots illustrate the strategy of gating for major T cell subsets defined by CD3^+^CD4^+^ (a), CD3^+^CD8^+^ (b), CD4^+^/45RA^+^ (c), and TCD4^+^/45RO^+^ (d) surface expressions in PBMC. All gates were based on isotype-matched control staining. Cumulative data showing the frequencies of total TCD4^+^ (e), total TCD8^+^ (f), TCD4^+^CD45RA^+^ (g), and TCD4^+^CD45RO^+^ (h) subpopulations within live-gated single cells. Each symbol represents an individual subject. Horizontal lines represent mean and standard error. ^∗^*p* < 0.05, ^∗∗^*p* < 0.005, ^∗∗∗^*p* < 0.0005, and ^∗∗∗∗^*p* < 0.00005.

Previous research has described alterations of lymphocyte phenotype and function in SCD patients, especially deregulated adaptive cell behavior, imbalanced T cell subsets, and alloimmunization [3, 18, 22, 23] To determine the frequencies of the major T cell subsets, we applied the staining panel to freshly isolated peripheral blood mononuclear cells (PB-MNC) from SCD, SCD/ON, and healthy control patients.  Table 3 and Figure 1 show the frequency for TCD4^+^ (T helper) and TCD8^+^ (T cytotoxic) coexpressing the CD3 marker. Compared with healthy controls, SCD and SCD/ON patients had a significantly lower median distribution of TCD4^+^ (41.98% (range 19.24-61.57) vs. 21.37% (range 5.67-47.5) and 30.04% (range 8.37-59.66), *p* = 0.01 and 0.05, respectively) and TCD8^+^ (24.02% (range 14.15-38.69) vs. 12.47% (range 4.83-23.77) and 14.57% (range 2.65-23.9), *p* = 0.01 and 0.005, respectively) cell subpopulations. There were no significant differences in these subsets between the SCD and SCD/ON groups. Figures 1(g) and 1(h) show TCD4^+^ effector cells (CD4^+^45RO^+^) and TCD4+ naïve cells (CD4^+^CD45RA^+^) frequencies in the control, SCD, and SCD/ON groups.

Frequencies of CD45RA^+^ within CD4^+^ T cell subpopulations were significantly higher in the control patients relative to both SCD (40.31% (range 20.79-59.84) vs. 10.78% (range 2.34-28.6), *p* = 0.0005) and SCD/ON (40.31% (range 20.79-59.84) vs. 12.60% (range 2.37-25.02), *p* = 0.0001) patients. Only SCD subjects had significantly lower median effector T cell proportions than the control subjects (21.62% (range 4.84-41.26) vs. 10.84% (range 4.33-16.08), *p* = 0.05). In sum, we found similar frequencies of major T cell subsets in the peripheral blood of SCD patients with or without osteonecrosis. Except for effector T cells in SCD/ON, control patients had significantly higher frequencies of T cell subsets in PB-MNCs in comparison to SCD, irrespective to the presence of osteonecrosis.

**Figure 2:**
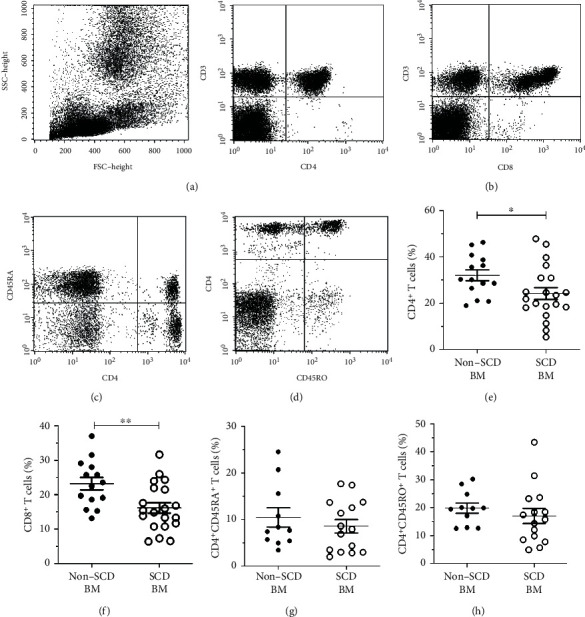
Relative frequency of major T cell populations in bone marrow mononuclear cells (BM-MNC) from SCD patients with osteonecrosis (SCD BM) and patients with osteonecrosis not related to SCD (non-SCD BM). Representative dot plots illustrate the strategy of gating for major T cell subsets defined by CD3^+^CD4^+^ (a), CD3^+^CD8^+^ (b), CD4^+^/45RA^+^ (c), and TCD4^+^/45RO^+^ (d) surface expressions in BM-MNC. All gates were based on isotype-matched control staining. TCD4^+^ and TCD8^+^ cells are lower in SCD BM. Cumulative data showing the frequencies of total TCD4^+^ (e), total TCD8^+^ (f), TCD4^+^CD45RA^+^ (g), and TCD4^+^CD45RO^+^ (h) subpopulations within live-gated single cells. Each symbol represents an individual subject. Horizontal lines represent mean and standard error. ^∗^*p* < 0.05; ^∗∗^*p* < 0.005.

Because many of the T lymphocytes migrate to the bone marrow and partly return later to the blood [24], we also analyzed the frequency of T cell subsets in the bone marrow aspirate of SCD patients affected by osteonecrosis (SCD BM) and patients with osteonecrosis not related to SCD (non-SCD BM).  Table 4 and Figure 2 show the distribution for the various lymphocyte subsets investigated in BM-MNC. We noted a higher frequency of TCD4^+^ and TCD8^+^ in non-SCD BM compared with SCD BM (32.01% (range 19.09-46.3) vs. 24.28% (range 5.41-47.82) and 23.20% (range 13.14-31.58) vs. 16.18% (range 6.4-31.68), *p* = 0.05 and *p* = 0.01, respectively). There were no significant differences in the frequency of other T subsets between groups.

### 3.3. Increased Frequency of IL4^+^TCD4^+^ Cells and Bifunctional IFN-*γ*^+^/IL4^+^ and IL-17^+^/IL4^+^CD4^+^T Cells in SCD and SCD/ON Compared to Healthy Control Patients

Next, we investigated functionally polarized TCD4^+^ cell subsets in SCD, SCD/ON, and healthy control patients. This was accomplished by simultaneously determining intracellular IFN-*γ*^+^ (representing Th1-like), IL4^+^ (representing Th2-like), and IL-17^+^ (representing Th17-like) expressions by flow cytometry after *in vitro* stimulation. Based on the distinctive patterns of intracellular cytokine expression, the percentage of mono- and bifunctional cells were assessed among the TCD4^+^ population. The spontaneous cytokine production of nonstimulated cells was minimal, and no significant differences were observed in the frequencies of TCD4^+^-expressing IFN-*γ*, IL4, and IL-17 between groups (data not shown).

**Figure 3:**
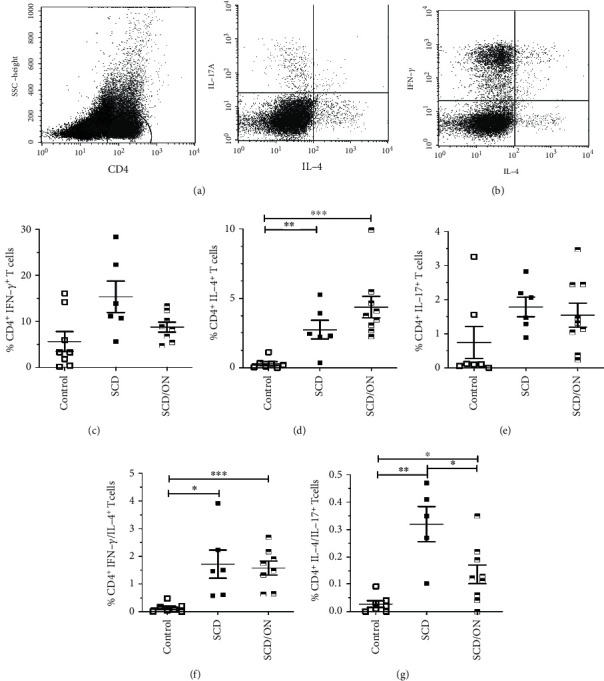
Increased frequency of IL4^+^CD4^+^ T cells and bifunctional IFN-*γ*^+^/ IL4^+^ and IL-17^+^/ IL4^+^CD4^+^ T cells in SCD and SCD/ON compared to healthy controls. Peripheral blood mononuclear cells from healthy donors (control), steady-state SCD, and SCD with osteonecrosis (SCD/ON) patients were culture stimulated with PMA/Io and stained for intracellular cytokine production. Representative dot plots illustrate intracellular staining for IFN-*γ*^+^/IL-4^+^ (a) and IL-4^+^/IL-17^+^ (b) in TCD4^+^ cells after stimulation. Relative frequency of IFN-*γ*^+^ (c), IL-4^+^ (d), IL-17^+^ (e), bifunctional IFN-*γ*^+^/IL-4^+^ (f), and IL-4^+^/IL-17^+^ TCD4^+^ (g) cells producing cytokines in relation to total TCD4^+^ cells in culture. Each symbol represents an individual subject. Horizontal lines represent mean and standard error. ^∗^*p* < 0.05, ^∗∗^*p* < 0.005, and ^∗∗∗^*p* < 0.0005.

After *in vitro* stimulation with PMA/ionomycin (PMA/Io) (polyclonal stimulation), a significantly higher frequency of IL4^+^TCD4^+^ cells in SCD and SCD/ON were detected in comparison to the control (0.19% (range 0.01-0.55) vs. 2.54% (range 1.98-4.41) and 0.19% (range 0.01-0.55) vs. 2.08% (range 0.57-4.15)) (*p* = 0.0016 and *p* = 0.0017, respectively) (Figure 3). When IFN-*γ*^+^ were analyzed, PB-MNC from the SCD and SCD/ON groups contained higher proportion of IFN-*γ*^+^ cells compared to the healthy control group; however, differences failed to reach statistical significance (*p* = 0.0593 and *p* = 0.1605, respectively) (Figure 3). Accordingly, the bifunctional IFN-*γ*^+^IL4^+^TCD4^+^ cells in SCD (1.72% (range 0.58-3.92)) and SCD/ON (1.57% (range 0.64-2.69)) were significantly higher compared to the control group (0.14% (range 0.00-0.48)) (*p* = 0.0264; *p* = 0.0006, respectively). In comparison to the healthy controls (0.02% (range 0.00-0.09)), significantly higher frequencies of bifunctional IL17^+^IL4^+^TCD4^+^ cells were noted in the SCD and SCD/ON groups (0.32% (range 0.10-0.47); 0.13% (range 0.00-0.35) (*p* = 0.0094 and *p* = 0.0162), respectively) (Figure 3(g)). The frequency of bifunctional TCD4^+^ cells coexpressing IL17^+^and IL4^+^ was significantly higher in SCD compared to the SCD/ON group (*p* = 0.0425). The increase in IL-4^+^TCD4^+^ subset frequency suggests a shift of CD4^+^ T cell response, corresponding to a Th2 phenotype.

**Figure 4:**
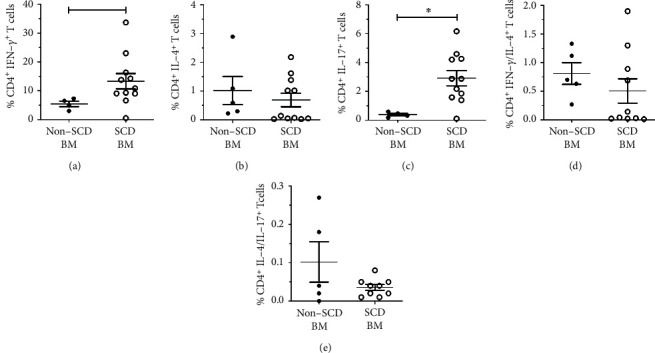
Increased frequency of IFN-*γ*^+^ CD4^+^ T and IL-17^+^CD4^+^ T cells in SCD BM compared to non-SCD BM patients. Bone marrow mononuclear cells (BM-MNC) from SCD patients with osteonecrosis (SCD BM) and patients with osteonecrosis not related to SCD (non-SCD BM) were culture stimulated with PMA/Io and stained for intracellular cytokine production. Relative frequency of IFN-*γ*^+^ (a), IL-4^+^ (b), IL-17^+^ (c), bifunctional IFN-*γ*^+^/IL-4^+^ (d), and IL-4^+^/IL-17^+^ TCD4^+^ (e) cells producing cytokines in relation to total TCD4^+^ cells in culture. Each symbol represents an individual subject. Horizontal lines represent mean and standard error. ^∗^*p* < 0.05, ^∗∗^*p* < 0.005, and ^∗∗∗^*p* < 0.0005.

We also investigated functional polarization of TCD4^+^ cell subsets in SCD and non-SCD BM based on intracellular cytokines IFN-*γ*, IL-4, and IL-17 expressions (Figure 4). The production of these cytokines was minimal in nonstimulated BM-MNC cultures (data not shown). When stimulated with PMA/ionomycin, the frequency of monofunctional IFN-*γ*^+^TCD4^+^ cells (Figure 4(a)) and IL-17^+^TCD4^+^ cells (Figure 4(c)) was increased in SCD BM compared to non-SCD BM (13.31% (range 0.55-33.68) vs. 5.45% (range 2.97-7.36) (*p* = 0.027, *p* < 0.0001), respectively). There was no difference in the relative frequency of IL-4^+^TCD4^+^ cells or bifunctional cells between SCD BM and non-SCD BM patients in culture-stimulated PMA/Io. The T cell immune response towards a cytokine secretion phenotype after stimulation indicated that the immune system of SCD patients has partially differentiated cells ready to assume Th1 and Th17 effector functions.

## 4. Discussion

Several SCD studies have reported evidences of abnormal immune cell counts and dysfunctional T lymphocyte response, but none of these previous works have investigated the immunological profile of SCD patients with osteonecrosis. In this small cross-sectional study, we demonstrated that lower frequency of circulating CD4^+^, CD8^+^, and naive T lymphocyte subsets were directly associated with SCD disease but not with osteonecrosis. After *in vitro* stimulation of PBMC, the percentage of IL4^+^TCD4^+^ cells coexpressing either IFN-*γ*^+^ or IL-17^+^ were significant higher in SCD and SCD/ON patients in comparison to control subjects. More importantly, the frequencies of IFN-*γ*^+^-producing and IL-17^+^-producing CD4^+^T cells in stimulated BM-MNCs were significantly higher in SCD/ON patients than controls patients with osteonecrosis not related to SCD. These results indicate that there are differences in peripheral and bone marrow T cell subsets in SCD patients with osteonecrosis that are able to assume a phenotype of cytokine secretion after stimulation. The changes observed on T cell responses could extend to inflammatory sites of bone complications, thereby contributing to the maintenance of inflammation and the pathophysiology of osteonecrosis. Given that previous studies have shown strong association between proinflammatory cytokines and osteonecrosis in SCD patients [6], our findings corroborate previous observations and support the notion that T cell may participate in the development of abnormal immune response and chronic inflammation in SCD patients with osteonecrosis.

In this context, we observed that the percentage of circulating CD4^+^, CD8^+^, and naïve TCD4^+^ cells were significantly reduced in SCD and SCD/ON patients. We also observed decreased percentage of CD4^+^ and CD8^+^ in the bone marrow of patient with osteonecrosis related to SCD in comparison to non-SCD patients. Many studies have correlated mild lymphopenia, elevated inflammation biomarkers, and poor outcomes in patients with SCD [4, 25]. It is worth nothing that the proportions of CD4^+^ T cells, naive CD4^+^ T cells, memory CD4^+^ T cells, memory CD8^+^ T cells, and CD4/CD8 ratio were profoundly reduced in the presence of splenic-associated abnormalities [26] or during vaso-occlusive crisis [22]. In fact, reduced peripheral CD4^+^ and Treg frequencies, as well as coexisting levels of both high and low Th1- and Th2-type cytokines in chronically transfused patients, suggested an underlying inflammatory state in SCD [27]. Together with reports of lymphopenia and reduced peripheral T cell levels in SCD patients [5, 18, 22], these findings suggest that T cells are attracted away from the blood and into the target site of inflammation. Recently, Nickel et al. described normalization of most CD4^+^T subpopulations and memory CD8^+^ T cells in SCD patients receiving immunosuppressant therapy to halt progression of chronic inflammation [28]. It must be pointed out that our SCD and SCD/ON patients were not under medication and they presented severe SCD biomarkers significantly different from the healthy control group. Overall, our results suggest that the decreased frequency of T cell subsets might be directly related to an underlying inflammatory state in SCD rather than osteonecrosis status.

In our study, there was an increased relative frequency of CD4^+^ T lymphocytes producing IL-4, IL-4/IL-17, or IL-4/INF-*γ* in samples from SCD and SCD/ON patients in comparison to healthy controls after stimulation with PMA/ionomycin. This finding corroborates previous reports that show an increased frequency of Th2 lymphocytes and elevated serum levels of IL-4 in SCD patients [22, 29]. The analysis of intracellular cytokine expression allowed a flexible identification of functional subtypes of T cells in contrast with the traditional classification defined by surface markers. Our finding suggests that SCD and SCD/ON patients show an increased subset of circulating T helper cells able to produce a broad spectrum of pro- and anti-inflammatory cytokines after a strong stimulus, such as PMA/ionomycin. In contrast, a significantly higher frequency of IL-17^+^, CD4^+^, and INF-*γ* CD4^+^ was observed in bone marrow T lymphocytes. There were no differences in the relative frequencies of IL4^+^CD4^+^ and bifunctional CD4^+^ T cells in the bone marrow of SCD BM patients compared with non-SCD BM patients. These results suggest that Th1 cells and Th17 are the main types of cellular immunity in the bone marrow of SCD patients, meaning that these increased T subsets in the bone marrow may contribute to the immune disorders in SCD patients with osteonecrosis. Recently, CD4^+^ T cells from steady-state SCD patients exhibit hallmarks of terminal T cell exhaustion and migration defects, including the expression of CTLA-4 and reduced expression of CCR7 [4]. Of relevance, emerging evidences have pointed toward RBC as dynamic reservoirs of cytokines that may affect immune function in SCD patients. Karsten et al. have shown that RBCs can store and release cytokines into the plasma, such as IFN-*γ*, IL-1*β*, IL-18, TNF-*α*, and several chemokines, including IL-8 and RANTES, suggesting that in hemolytic conditions, such as sickle cell disease, RBC may play downstream effects on neighboring cells and may have a critical role in modulating T cell behavior, including different subsets of T cells [30]. Indeed, IL6 and IL-10 frequently produced by SCD patients could also be associated with the alterations in immune profile and functions [6], although the reason for these particular phenotypes is not fully understood.

Bone homeostasis is regulated by the immune system [31–34]. In our study, we demonstrated that polyclonal stimulation of BM-MNC induced an increased frequency of CD4^+^IFN-*γ*^+^ and CD4^+^IL-17^+^ in SCD BM compared to non-SCD BM patients. Previous studies have shown that activated T cells play an important role in bone health and disease. Especially, Th17 cells have a great influence on bone metabolism by secreting characteristic soluble factor that induces osteoclastogenesis [35]. In contrast, effector T cell production of IFN-*γ* strongly suppresses bone resorption by interfering with the RANKL/RANK signaling pathway [36]. It has been shown that increased serum levels of IL-17A and IFN-*γ* are present in patients with steroid-related osteonecrosis [37]. All together, these results suggest that Th17 and Th1 cells may have a role in the pathogenesis of osteonecrosis in sickle cell disease. The combined analysis of the data herein presented, together with the existing information in the literature, provides clues to a better understanding of the pathophysiology of osteonecrosis related to SCD and opens perspectives to the development of alternative therapies for this disease.

It is conceivable that personalized cytokine blocking therapy specifically designed according to the predominant profile of functional T cells will be effective in helping restore the immunologic balance in each SCD patient. IL-17-producing and IFN-*γ*-producing T lymphocytes seem to play a prominent role in pathophysiology of osteonecrosis related to SCD and may represent a potential target for therapy. In fact, it is possible that cytokine-targeted and personalized therapy may contribute to improving the balance of effector immune response, avoiding or minimizing the damage caused by the unbalanced immune response.

Our study has some limitations. First, this study was cross-sectional and thus only involved a single timepoint for each of the enrolled patients. A prospective longitudinal study is necessary to fully evaluate the causal relationship between osteonecrosis onset or progression and the T cell subset changes in SCD patients. Second, although the changes in lymphocyte subset numbers and the functional status of many of the CD4+ T cells were investigated, it cannot be assumed to reflect functional immune deviation. To overcome this limitation, antigen-specific tests of immune activation will be important to add to further *in vitro* studies. Finally, increasing sample size should improve statistic power and reproducibility on repeated measured outcomes. Future studies that include more SCD patients both at steady state as well as with osteonecrosis would shed light on the impact of this complication on the immune manifestations of SCD.

## 5. Conclusion

In conclusion, this study has described that SCD patients with or without ON presented significantly reduced TCD4^+^, TCD8^+^, and TCD4^+^ naïve cell frequencies in PB-MNC and increased frequency of circulating CD4^+^T cells able to simultaneously produce IFN-*γ*^+^/IL4^+^ and IL-17^+^/IL4^+^ compared to healthy controls. Conversely, there was an increased frequency of either IFN-*γ*^+^ or IL-17^+^CD4^+^T cells in stimulated BM-MNC of SCD patients with osteonecrosis. This finding suggests that the bone marrow of patients with SCD show an increased subset of T helper cells able to produce a broad spectrum of proinflammatory cytokines after a strong stimulus, such as PMA/Io. This increased CD4^+^T subset may extend to inflammatory sites of target organs and may contribute to the immune disorders in SCD patients with osteonecrosis.

## Figures and Tables

**Table 1:** Demographic and hematologic profiles for peripheral blood, from the control, SCD, and SCD/ON groups.

	Control	SCD	SCD/ON	*p* value		
				Control vs. SCD	Control vs. SCD/ON	SCD vs. SCD/ON
*N* (%)	19 (44.2)	9 (20.9)	15 (34.9)			
Age (y)	31 (18-49)	30 (19-47)	31 (18-55)			
Male (%)	52.63	88.88	46.66			
WBC (10^3^/mm^3^)	6.9 (3.5-9.3)	7.4 (4.5-13.3)	9.6 (5.0-13.1)	0.182	0.0008^∗∗∗^	0.546
Lym (%)	37.1 (9.5-54.5)	40.5 (24.2-57.8)	32.8 (26.2-38.7)	0.443	0.272	0.083
RBC (10^6^/mm^3^)	5.4 (3.3-7.8)	2.9 (2.5-5.1)	3.7 (2.3-5.5)	0.003^∗∗^	0.002^∗∗^	0.469
Hgb (g/dL)	14.6 (9.2-21.0)	10.9 (8.4-13.5)	11.8 (8.6-16.2)	0.009^∗∗^	0.050^∗^	0.289
Hct (%)	44.6 (28.4-69.3)	31.3 (23.2-41.0)	33.3 (18.2-50.8)	0.007^∗∗^	0.002^∗∗^	0.666
MCV (fL)	86.2 (76.7-91.0)	93.6 (79.9-101.4)	91.2 (79.2-117.5)	0.010^∗^	0.165	0.228
MCH (pg)	27.3 (18.9-34.4)	32.2 (23.7-37.0)	31.6 (27.4-37.3)	0.035^∗^	0.013^∗^	0.253
RDW (%)^a^	13.4 (11.8-17.3)	18.3 (17.0-19.4)	14.5 (11.4-18.0)	<0.0001^∗∗∗∗^	0.1007	0.0001^∗∗∗^
Plat (10^3^/*μ*L)	196.0 (107.0-472.0)	241.0 (68.0-451.0)	257.5 (55.0-532.0)	0.950	0.370	0.931

Results are presented as median (min–max), except where noted otherwise. WBC: white blood cell; Lym: lymphocytes; RBC: red blood cells; Hgb: hemoglobin; Hct: hematocrit; MCV: mean corpuscular volume; MCH: mean corpuscular hemoglobin; RDW: red cell distribution width; and Plat: platelets. Significance values are presented as ^∗^*p* < 0.05, ^∗∗^*p* < 0.005, ^∗∗∗^*p* < 0.0005, and ^∗∗∗∗^*p* < 0.00005.

**Table 2:** Demographic and hematology profiles for the bone marrow aspirate, from the SCD and non-SCD groups.

	Non-SCD BM (*N* = 18)	SCD BM (*N* = 24)	*p* value
Age (y)	36 (16-59)	25.7 (9-57)	
Male (%)	61.1	50	
WBC (×10^3^/mm)	7.8 (5.0-24.5)	12.8 (6.2-31.9)	0.081
Lym (%)	34.5 (21.9-48.4)	36.9 (5.2-55.4)	0.450
RBC (×10^6^/mm^3^)	4.0 (2.1-6.3)	3.1 (1.7-5.2)	0.005^∗∗^
Hgb (g/dL)	12.8 (7.1-17.8)	10.3 (6.7-13.5)	0.026^∗^
Hct (%)	36.3 (19.9-57.2)	31.7 (18.0-45.0)	0.058
MCV (fL)	88.9 (78.2-95.1)	103.9 (71.0-135.5)	0.001^∗∗^
MCH (pg)	32.2 (23.3-37.9)	35.6 (25.7-46.1)	0.217
RDW (%)	12.5 (10.2-16.3)	14.8 (10.9-19.4)	0.001^∗∗^
Plat (10^3^/*μ*L)	8.0 (6.5-10.2)	8.7 (7.8-9.8)	0.037^∗^
CD34^+^CD45^low^ (%)	0.6 (1.3-0.2)	0.5 (1.2-0.2)	0.401

^a^Results are presented as median (min–max), except where noted otherwise. WBC: white blood cell; Lym: lymphocytes; RBC: red blood cells; Hgb: hemoglobin; Hct: hematocrit; MCV: mean corpuscular volume; MCH: mean corpuscular hemoglobin; RDW: red cell distribution width; and Plat: platelets. Significance values are presented as *p*; ^∗^*p* < 0.05, ^∗∗^*p* < 0.005, ^∗∗∗^*p* < 0.0005, and ^∗∗∗∗^*p* < 0.00005.

**Table 3:** Lymphocyte frequencies (%) in PB-MNC.

	Control	SCD	SCD/ON	*p* value		
				Control vs. SCD	Control vs. SCD/ON	SCD vs. SCD/ON
TCD4^+^	42.0 (19.2-61.6)	21.4 (5.7-47.5)	30.0 (8.4-59.7)	0.0074^∗∗^	0.0442^∗^	0.2854
TCD8^+^	24.0 (14.1-38.7)	12.5 (4.8-23.7)	14.6 (2.6-23.9)	0.0085^∗∗^	0.0020^∗∗^	0.5962
TCD4^+^/45RO^+^	21.6 (4.8-41.3)	10.8 (4.3-16.1)	15.5 (4.6-25.6)	0.0154^∗^	0.1541	0.1892
TCD4^+^/45RA^+^	40.3 (20.8-59.4)	10.8 (2.3-28.6)	12.60 (2.4-25.0)	0.0003^∗∗∗^	<0.0001^∗∗∗∗^	0.8749

All values are presented in median. Significance values are presented as ^∗^*p* < 0.05, ^∗∗^*p* < 0.005, ^∗∗∗^*p* < 0.0005, and ^∗∗∗∗^*p* < 0.00005.

**Table 4:** Lymphocyte frequencies (%) in BM-MNC.

	Non-SCD BM	SCD BM	*p* value
TCD4^+^	32.0 (19.09-46.3)	24.28 (5.41-47.82)	0.0332^∗^
TCD8^+^	23.20 (13.14-31.58)	16.18 (6.4-31.68)	0.0065^∗∗^
TCD4/45RO^+^	19.81 (12.61-30.27)	17.00 (4.94-43.4)	0.3965
TCD4/45RA^+^	7.68 (3.47-24.52)	8.57 (2.01-17-68)	0.5065

All values are presented in mean. Significance values are presented as ^∗^*p* < 0.05, ^∗∗^*p* < 0.005, ^∗∗∗^*p* < 0.0005, and ^∗∗∗∗^*p* < 0.00005.

## Data Availability

The data used to support the findings of this study are available from the corresponding author upon request.
